# Effects of nutrition management combined with specialized nursing on nutritional status, immune function and wound healing after joint replacement

**DOI:** 10.12669/pjms.41.4.10524

**Published:** 2025-04

**Authors:** Haimei Ren, Liang Jiang, Yibin Zhou, Xiaohui Qi, Jingjie Cui

**Affiliations:** 1 Haimei Ren Department of Orthopaedics, Affiliated Hospital of Hebei University, Baoding 071000, Hebei, China; 2 Liang Jiang Department of Orthopaedics, Affiliated Hospital of Hebei University, Baoding 071000, Hebei, China; 3 Yibin Zhou Department of Orthopaedics, Affiliated Hospital of Hebei University, Baoding 071000, Hebei, China; 4 Xiaohui Qi Department of Pulmonary and Critical Care Medicine, Affiliated Hospital of Hebei University, Baoding 071000, Hebei, China; 5 Jingjie Cui Department of Orthopaedics, Affiliated Hospital of Hebei University, Baoding 071000, Hebei, China

**Keywords:** Nutrition management, Joint replacement, Wound healing, Nutritional status, Immune function

## Abstract

**Objective:**

To explore the effects of nutrition management combined with specialized nursing on nutritional status, immune function and wound healing of patients after joint replacement.

**Methods:**

Medical data of patients who underwent hip/knee replacement (n= 80) in Affiliated Hospital of Hebei University from June 2021 to December 2023 were collected for retrospective analysis. The included patients were divided into an observation group (special nutrition management + specialized nursing) and a control group (routine nutrition guidance + specialized nursing) according to different nutrition managements, n=40 each group. Moreover, nutritional status, immune function, wound healing and nursing satisfaction were compared between the two groups.

**Results:**

There was no significant difference in nutritional status or immune function between the observation group and the control group before the operation, while obvious improvements in nutritional status and immune function were observed in both groups at discharge (*p*< 0.05). In addition, significantly higher levels of nutritional indicators (Hb, Alb, PA, TF and RBC) and immunoglobulin (IgG, IgA and IgM) were found in the observation group when compared with the control group (all *p*< 0.05); and the incidences of wound dehiscence and local skin necrosis in the observation group were remarkably lower than those in the control group (all *p*< 0.05). In terms of the nursing satisfaction, the observation group presented significantly-higher patient satisfaction on nursing than the control group (95.00% vs. 80.00%).

**Conclusion:**

Nutrition management combined with specialized nursing may achieve significant improvements in nutritional status, immune function, wound healing and nursing satisfaction of patients after joint replacement.

## INTRODUCTION

Artificial joint replacement was considered one of the primary treatments for joint diseases, and hip and knee replacement was more frequently performed in clinical practice, with the 10-years success rate being 90% or above.^1^ With the aggravation of population aging in China, there was an increasing number of elderly people who underwent hip and knee replacement, and significant improvements have been achieved in hip and knee mobility and quality of life.^2^,^3^ However, it has been demonstrated that the postoperative recovery of patients who underwent hip and knee replacement might be seriously affected due to large trauma, enhanced physical stress reaction caused by postoperative pain and the occurrence of postoperative complications.^4^-^6^ Additionally, a previous study reported that the elderly had poor surgical tolerance to hip and knee replacement, decreased gastrointestinal motility and loss of appetite occurred after the operation would lead to reduced nutrient intake, prolonged wound healing time and extended recovery cycle.^7^

Generally speaking, nutrition intervention in elderly patients has failed to be explicitly stipulated in traditional clinical nursing, and the importance of a nutritional diet in postoperative recovery was also poorly appreciated.^8^,^9^ Fortunately, people have gradually started to pay more attention to postoperative nutrition, rehabilitation exercise and psychological regulation at present; and postoperative nutrition management has also been valued by various clinical disciplines with the development of nutrition and nursing science. Therefore, the study was conducted to explore the effects of nutrition management combined with specialized nursing on the recovery of the elderly after joint replacement.

## METHODS

This was a retrospective study. Eighty elderly patients who underwent hip/knee replacement in Affiliated Hospital of Hebei University from June 2021 to December 2023 were selected as the subjects. Patient data were collected from the electronic medical record system of our hospital, including baseline characteristics, medical history, physical examination and laboratory examination. The included subjects were randomly divided into observation group (n= 40) and control group (n= 40). There were 21 males and 19 females in the observation group with a mean age of 67.65 ± 5.44 (60-78) years old and 23 males and 17 females in the control group with a mean age of 69.25 ± 5.55 (60-79) years old, respectively. No significant difference was found in baseline information between the observation group and the control group (*p>* 0.05), indicating that the obtained data were comparable (Table-I).

**Table-I:** Comparative analysis of the general information of patients in the observation group and the control group (*χ̅*±*S*, n = 40).

Index	Observation group	Control group	t/c^2^	p
Age (years)	67.65 ± 5.44	69.25 ± 5.55	-1.302	0.197
Male (n, %)	21 (52.50%)	23 (57.50%)	0.202	0.822
BMI (kg/m^2^)	26.20 ± 2.67	26.35 ± 2.21	-0.273	0.785
Hip/knee replacement (n)	18/22	21/19	0.450	0.502

*Notes:* BMI, body mass index.

### Ethical Approval:

The study was approved by the Institutional Ethics Committee of Affiliated Hospital of Hebei University (No: HDFYLL-KY-2024-077; Date: September 25,2023), and written informed consent was obtained from all participants.

### Inclusion criteria:

- Patients with age ≥ 60 years old.
- Patients without mental illness.
- Patients with complete clinical data.
- Patients who underwent hip and knee replacement.
- The informed consent was voluntarily signed by patients or their families.

### Exclusion criteria:

- Patients with malignant tumors.
- Patients complicated with dysfunction or failure of important organs.
- Patients with diseases affecting wound healing, such as diabetes mellitus, etc.

### Nursing intervention:

Specialized nursing was carried out on patients in both groups with the following details:

### Psychiatric nursing:

informed the patient and family members about procedures of joint replacement, postoperative rehabilitation and possible complications before the operation, as well as the positive significance of maintaining a positive and good mental state for achieving a good prognosis; gone to the bedside immediately after the patient woke up to communicate with him/her, informed the patient about operation details and followed-up rehabilitation measures, and encouraged the patient to actively cooperate with the medical staff for following treatments; and asked specialists for consultation and treatment in time for patients with obvious emotional disorders in the perioperative period.

### Drainage nursing:

properly fixed the drainage tube and recorded the volume, character and color of drainage fluid in detail; carried out rinse with sterile normal saline in case of inadequate drainage; informed the sickbed managing physician in time if the color of drainage fluid was abnormal; and controlled the indwelling time of the drainage tube not exceeding seven days and strictly perform the aseptic operation when replacing and removing the drainage tube.

### Specialized Nursing Care:

Complication nursing: asked the patient to clean the mouth, percuss the back and turn over every day after the operation, and encouraged the patient to cough and expectorate actively; carried out nasal feeding first to avoid pulmonary infection for patients with consciousness disorder; and changed the drainage pack connected with the urethral catheter every day, and disinfected the vulva and urethra with iodophor at the same time.

### Nutrition management:

Nutrition management was carried out in both groups with the following steps:

### Control group:

conducted admission education, emphasized all the precautions, provided brochures, etc.; gave the patients routine dietary guidance according to the doctor’s advice, and asked the patient to eat more food rich in high-quality protein and reduced the intake of greasy food by considering patient’s condition and basic diseases.

### Observation group:

Assessed the nutritional status of patients by the professional dietitian considering clinical examination results, formulated a diet plan based on assessment results and dietetic contraindications, gave a detailed explanation of the importance of a nutritious diet to patients by the dietitian from a professional perspective, answered the questions raised by patients, relieved the doubts of the patients, and then made the diet clear to the family members by the medical staff according to the doctor’s orders. For example, during the intervention period, when patients developed loss of appetite and indigestion due to the diet, asked patients and family members to communicate with the dietitian in time to adjust the diet plan or adopt other methods.

### Observation indicators:

### Nutritional status and immune function:

One day before discharge, fasting venous blood samples were collected in the morning for further detection. The levels of hemoglobin(Hb), albumin(Alb), prealbumin(PA), transferrin(TF) and red blood cell(RBC) were compared between the two groups. The serum levels of immunoglobulin (IgG, IgA and IgM), the levels of T-lymphocyte subsets (CD3^+^, CD4^+^ and CD8^+^) and the ratio of CD4^+^/CD8^+^ were measured by an immunoassay analyzer. Blood samples were collected in all cases under fasting condition in the morning.

### Wound healing:

Wound drainage device placement time, wound drying time and suture removal time of patients in the observation group and the control group were recorded, and the incidences of poor wound healing events were compared between the two groups, including wound dehiscence, wound infection, local skin necrosis, etc.

### Nursing satisfaction:

Nursing satisfaction questionnaires were distributed to patients and their families on the day of discharge, including the following contents: nursing quality, nursing comfort degree, environment, etc. Full marks were set at 100 points: 0-59: unsatisfied; 60-84: satisfied; and 85-100: very satisfied. Nursing satisfaction = (basically satisfied + very satisfied)/the number of cases × 100%.

### Statistical analysis:

SPSS22.0 was applied for data analysis. Measurement data were expressed as mean ± standard deviation (*χ̅*±*S*), and comparisons before and after operation were performed with the use of *t*-test. The significance level α was set to 0.05 and the confidence interval was 95%. Two independent sample t-test was used for comparison between groups, and paired t-test was used to analyze data within groups. Enumeration data were expressed as n(%), and c^2^ was adopted for comparisons between groups. *P*< 0.05 indicated that the difference was statistically significant.

## RESULTS

There was no significant difference in the levels of Hb, Alb, PA, TF and RBC between the two groups before treatment (*p>* 0.05); while the levels of Hb, Alb, PA, TF and RBC in the two groups at discharge were obviously higher than those before the operation, and significantly higher levels of Hb, Alb, PA, TF and RBC were observed in the observation group when compared with the control group at discharge (*p<* 0.05) (Table-II).

**Table-II:** Comparisons in nutrition indicators between the two groups (*χ̅*±*S*, n= 40).

Indicators		Observation group	Control group	t	p
Hb (g/L)	Before treatment	105.93 ± 7.69	103.50 ± 5.94	1.578	0.119
	After treatment	117.03 ± 7.28	111.73 ± 6.07	3.536	0.001
Alb (g/L)	Before treatment	38.27 ± 4.97	38.19 ± 5.87	0.066	0.948
	After treatment	48.43 ± 3.42	45.81 ± 5.02	2.723	0.008
PA (mg/L)	Before treatment	233.92 ± 34.46	229.58 ± 31.18	0.592	0.556
	After treatment	289.89 ± 33.01	268.56 ± 32.15	2.927	0.004
TF (mmol/L)	Before treatment	40.95 ± 1.27	40.28 ± 1.84	1.913	0.059
	After treatment	53.22 ± 1.54	51.05 ± 1.98	5.471	0.000
RBC (10^12^/L)	Before treatment	4.21 ± 0.57	4.32 ± 0.49	-0.901	0.370
	After treatment	4.81 ± 0.48	4.59 ± 0.48	2.041	0.045

*Notes:* Hb, hemoglobin; Alb, albumin; PA, prealbumin; TF, transferrin; RBC, red blood cell.

There was no significant difference in the levels of IgG, IgA and IgM between the two groups before treatment (*p>* 0.05); while the levels of IgG, IgA and IgM in the two groups were evidently higher at discharge than those before the operation, and significantly higher levels of IgG, IgA and IgM were found in in the observation group when compared with the control group (*p<* 0.05) (Table-III).

**Table-III:** Comparative analysis of immunoglobulin levels before and after treatment between the two groups (*χ̅*±*S*, n= 40).

Group		Before treatment	At discharge	t	p
IgG (g/L)	Observation group	7.64 ± 2.48	13.87 ± 4.44	-7.744	0.000
	Control group	7.84 ± 2.45	11.38 ± 4.50	-4.368	0.000
	*t*	-0.353	2.485		
	*P*	0.725	0.015		
IgA (g/L)	Observation group	1.36 ± 0.27	2.85 ± 0.76	-11.660	0.000
	Control group	1.33 ± 0.31	2.32 ± 0.48	-10.984	0.000
	*t*	0.493	3.751		
	*P*	0.623	0.000		
IgM (g/L)	Observation group	1.41 ± 0.66	2.78 ± 0.64	-9.417	0.000
	Control group	1.39 ± 0.71	2.42 ± 0.52	-7.430	0.000
	*t*	0.131	2.738		
	*P*	0.896	0.008		

We found no significant difference in the levels of CD3^+^, CD4^+^ and CD8^+^ and the ratio of CD4^+^/CD8^+^ between the two groups before treatment (*p>* 0.05); and there were significantly increased levels of CD3^+^ and CD4^+^, an obviously higher ratio of CD4+/CD8+ and an evidently decreased level of CD8 + in the observation group at discharge in comparison with the control group (all *p<* 0.05) (Table-IV).

**Table-IV:** Comparative analysis of T-lymphocyte subset levels between the two groups before and after treatment (*χ̅*±*S*, n=40).

Indicators		Observation group	Control group	t	p
CD3+(%)	Before treatment	42.82 ± 6.74	42.94 ± 6.21	-0.079	0.937
	After treatment	48.69 ± 6.55	43.47 ± 6.22	3.657	0.000
CD4+(%)	Before treatment	27.44 ± 4.04	27.61 ± 3.74	-0.201	0.841
	After treatment	38.32 ± 4.62	34.12 ± 4.77	3.994	0.000
CD8+(%)	Before treatment	21.50 ± 3.70	21.75 ± 3.69	-0.303	0.763
	After treatment	22.08 ± 3.71	23.88 ± 4.23	-2.031	0.046
CD4+/CD8+	Before treatment	1.28 ± 0.10	1.28 ± 0.07	0.277	0.782
	After treatment	1.76 ± 0.18	1.45 ± 0.16	8.016	0.000

After the operation, the drainage device placement time, the wound drying time and the wound healing time in the observation group were significantly shorter than those in the control group (*p<* 0.05). Wound infection was observed in both groups but without a significant difference in the incidence of wound infection between groups (2.50% vs 7.50%, *p>* 0.05). The observation group had obviously lower incidences of wound dehiscence and local skin necrosis than the control group (*p<* 0.05) (Table-V).

**Table-V:** Comparison of would conditions between the two groups.

Group	Drainage device placement time (day)	Wound drying time (day)	Suture removal time (day)	Wound dehiscence [n (%)]	Wound infection [n (%)]	Local skin necrosis [n (%)]
Observation group (*n*= 40)	2.65 ± 0.66	3.80 ± 0.69	13.80 ± 0.72	2 (5.00%)	1 (2.50%)	1 (2.50%)
Control group (*n*= 40)	3.00 ± 0.72	4.15 ± 0.80	15.10 ± 0.78	8 (20.00%)	3 (7.50%)	6 (15.00%)
*t/c^2^*	-2.270	-2.096	-7.741	4.114	0.641	3.914
*p*	0.026	0.039	0.000	0.043	0.423	0.048

At discharge, the patient satisfaction with nursing care in the observation group was significantly higher compared with the control group (95.00% vs. 80.00%, c^2^ = 4.114, *P* = 0.043) (Table-VI).

**Table-VI:** Comparison of patient satisfaction with nursing care between the two groups.

Group	Very satisfied [n (%)]	basically satisfied [n (%)]	Unsatisfied [n (%)]	Satisfaction [n (%)]
Observation group (*n* = 40)	21 (52.50%)	17 (42.50%)	2 (5.00%)	38 (95.00%)
Control group (*n* = 40)	14 (35.00%)	18 (45.00%)	8 (20.00%)	32 (80.00%)
*c^2^*				4.114
*p*				0.043

## DISCUSSION

High protein and high-calorie foods were selected in the present study to effectively improve patient energy and nutrient element intake on the basis of scientific and reasonable nutrition management.^10^ Furthermore, timely secondary adjustment to the formulated recipes was required for different patients, so as to achieve the effect of giving consideration to the treatment compliance and nutritional needs of patients. Hb, Alb, PA, TF and RBC, major components in the blood, were widely accepted indicators used to directly determine the nutritional level of patients. Our results showed that Hb, Alb, PA, TF and RBC in the observation group performed with nutritional management were significantly higher than those in the control group(p<0.05), intuitively revealing that nutrition management could effectively optimize the nutritional status of patients.

In nutrition management, a comprehensive assessment was performed on the nutritional status of patients by dietitians and primary nurses, and then scientific dietary recipes before and after operation were formulated in combination with laboratory examination indicators^11^,^12^; meanwhile, dietitians and primary nurses were asked to conduct timely cause analysis and countermeasure for patients with poor feeding state by strengthening the nutrition nursing awareness of clinical nursing staff, so as to prevent nursing work from becoming a mere formality. Traverso G et al,^13^ found that the one-year survival rate of patients with normal Hb was significantly higher than that of patients with decreased Hb, and decreased Alb might be considered an independent prognostic factor for predicting patient death.

Attributed to the advent of an aging society in China, there was a growing number of patients suffering from severe hip or knee osteoarthritis. Artificial joint replacement was generally believed as one of the primary means for the treatment of advanced degenerative bone and joint diseases, and the majority of patients with hip or knee osteoarthritis tended to receive an artificial joint replacement as an approach to alleviate pain and improve quality of life.^14^ As demonstrated by related studies, there was an increased body demand for proteins due to surgical trauma and postoperative inflammatory reaction, and acute-phase protein would be preferentially synthesized by the liver in the stress state, resulting in reduced production of Alb.^15^

Hypoproteinemia could bring damage to cell-mediated immune function, and the decrease in serum protein content was suggested to directly cause the reduction of enzymes for antibody synthesis, as well as lowered enzymatic activity, thus affecting both the cell immune system and the humoral immune system.^16^ As for T-lymphocyte subsets representing cellular immune function, it’s well known that the level of CD4^+^ was positively correlated with the overall state of cellular immunity, and CD8+ cyto-toxic T lymphocytes would be produced in large quantities in case of toxin invasion and decline in immune function. With respect to immunoglobulin acting as the main executor of humoral immune function, there was a close relationship between the contents of IgG, IgA and IgM and the humoral immune function.

A previous study has reported that the elderly patients had remarkably lower contents of IgG, IgA, IgM and other immunoglobulins in comparison with normal people, and obvious characteristics of abnormal T lymphocyte subset content were found, indicating that both the cell immune system and the humoral immune system were affected.^17^ Regarding immunoglobulins and T-lymphocyte subsets, our findings showed that there were significantly higher contents of IgG, IgA and IgM and obviously increased levels of CD3^+^and CD4^+^, significantly decreased level of CD8^+^ and an evidently higher ratio of CD4^+^/CD8^+^ in the observation group when compared with the control group, suggesting that nutrition management combined with specialized nursing had an effect of optimizing the cellular and humoral immune function of patients at the same time.

The possible mechanism to explain our findings may be that nutrition management combined with specialized nursing had the capabilities of supplementing exogenous nutrients in a timely, reasonable and whole-course manner, improving serum protein contents, reducing the level of catabolic hormones in the body and reducing protein consumption of the body; moreover, nutrition management combined with specialized nursing could increase enzymes required for antibody synthesis and improve enzyme activity, thus finally promoting the rapid recovery of the immune system of the body.

Aseptic inflammatory reaction, soft tissue and vascular regeneration and epithelial tissue repair were involved in the pathophysiological process of wound healing, and poor wound healing would be observed when any stage of the above-mentioned process was interfered with by adverse factors. Additionally, previous studies also have found that malnutrition might lead to the decline of postoperative wound healing ability. It has been evidenced that the elderly patients were more likely to show obviously poor wound healing process and quality compared with the young patients due to associated diseases and poor nutritional status.^18^

In the present study, comparisons of the wound healing in the two groups showed that the drainage device placement time, wound drying time and wound healing time in the observation group were less than those in the control group; and significantly lower incidence of poor healing such as wound dehiscence, wound infection and local skin necrosis were found in the observation group in comparison with the control group, which was consistent with the results of Guo SJ^19^, suggesting that nutrition management and intervention could effectively promote postoperative wound healing and reduce the incidence of poor wound healing in elderly patients, so as to improve the overall rehabilitation quality of patients.

### Limitations:

Firstly, the number of enrolled clinical cases was small. Secondly, the inclusion and exclusion criteria established in the study failed to completely avoid other risk factors affecting wound healing. In view of this, more patients should be included and the inclusion and exclusion criteria should be improved in future studies to further validate the findings of this study.

## CONCLUSIONS

The elderly patients had decreased physical function, while targeted nutrition management and specialized nursing may have significant clinical advantages on the recovery of the elderly after joint replacement, characterized by the effects in providing patients with good nutrition supply, ensuring the nutritional support required by patients during recovery, improving the immune function, shortening the rehabilitation cycle, promoting wound healing and improving nursing satisfaction.

### Authors’ Contributions:

**HR:** Literature search, Carried out the studies, data collection, drafted the manuscript, responsible and accountable for the accuracy or integrity of the work.

**LJ, XQ, JC** and **YZ:** Performed the statistical analysis and participated in its design. Critical Review.

All authors have read and approved the final manuscript.
